# Longitudinal Verification of Post-Nuclear Accident Food Regulations in Japan Focusing on Wild Vegetables

**DOI:** 10.3390/foods11081151

**Published:** 2022-04-15

**Authors:** Minoru Osanai, Tomuhiro Noro, Shonosuke Kimura, Kohsei Kudo, Shota Hosokawa, Megumi Tsushima, Ryoko Tsuchiya, Kazuki Iwaoka, Ichiro Yamaguchi, Yoko Saito

**Affiliations:** 1 Department of Radiation Science, Hirosaki University Graduate School of Health Sciences, Hirosaki 036-8564, Aomori, Japan; kohsei@hirosaki-u.ac.jp (K.K.); shosokawa@hirosaki-u.ac.jp (S.H.); tmegumi@hirosaki-u.ac.jp (M.T.); yokosait@hirosaki-u.ac.jp (Y.S.); 2 Department of Radiological Technology, Hirosaki University School of Health Sciences, Hirosaki 036-8564, Aomori, Japan; h18m2232@hirosaki-u.ac.jp (T.N.); h18m2214@hirosaki-u.ac.jp (S.K.); 3 Department of Nursing, Hirosaki University Graduate School of Health Sciences, Hirosaki 036-8564, Aomori, Japan; tsuchiya@hirosaki-u.ac.jp; 4 Department of Radiation Regulatory Science Research, National Institute of Radiological Sciences, National Institutes for Quantum Science and Technology, Inage 263-8555, Chiba, Japan; iwaoka.kazuki@qst.go.jp; 5 Department of Environmental Health, National Institute of Public Health, Wako 351-0197, Saitama, Japan; yamaguchi.i.aa@niph.go.jp

**Keywords:** radionuclides, standard limits, food monitoring test, Fukushima Dai-ichi Nuclear Power Plant accident, food regulation, food safety, internal exposure dose, risk assessment, wild vegetables

## Abstract

Focusing on the importance of wild vegetables for local residents, this study aims to validate the effects of food regulations under the current criteria (e.g., 100 Bq/kg for general foods) established approximately a year after the Fukushima Dai-ichi Nuclear Power Plant accident. Over 2,500,000 monitoring tests were performed under the criteria until fiscal year (FY) 2020. We estimated changes in internal exposure dose using test results. The effective dose was estimated using the radioactive concentration randomly sampled from the results, food intake, and dose conversion factor. As a new attempt, dose estimation reflecting the intake of wild vegetables that may have irreplaceable value for local residents was conducted. The median, 95th, and 99th percentile of the estimated dose without reflecting the wild vegetables’ intake were 0.0485, 0.183, and 10.6 mSv/year, respectively, in the estimation with all test results (no regulation) and 0.0431, 0.0786, and 0.236 mSv/year, respectively, in the estimation with results within the standard limits (regulated) in FY2012. These doses decreased with time. Although estimated doses with or without the reflection of wild vegetables’ intake were similar, estimation that is more plausible is possible, particularly for a high percentile, by reflecting the wild vegetables’ intake. Radiation doses (regulated) were significantly less than 1 mSv/year in different FYs. In Japan, food regulation measures benefit food safety.

## 1. Introduction

The Great East Japan Earthquake and tsunami on 11 March 2011 caused the Fukushima Dai-ichi Nuclear Power Plant (FDNPP) accident. Due to the accident, radionuclides were released into the environment [1] and detected in foodstuff. In Japan, the Ministry of Health, Labour, and Welfare (MHLW) established provisional regulatory values for radioactive substances in foods on 17 March as an urgent measure [2,3]. The values for radioactive cesium were set as the radionuclide concentration for radioactive cesium based on an effective dose of 5 mSv/year. The value was 200 Bq/kg for the categories of drinking water and milk/dairy products and 500 Bq/kg for that of vegetables, grains, meat, eggs, and fish. To deal with long-term situations, the present standard limits (current criteria) were established on 1 April 2012, through discussions in the Pharmaceutical Affairs and Food Sanitation Council [4]. The current criteria for radioactive cesium (sum of cesium-134 (Cs-134) and cesium-137 (Cs-137)) were established based on an effective dose of 1 mSv/year, which is in harmony with a reference level adopted by the Codex Alimentarius Commission (CAC) based on publication 82 of the International Commission on Radiological Protection (ICRP) [5,6,7,8,9]. The regulatory values of the current criteria are presented in Table 1.

Although Cs-134 and Cs-137 were certainly the dominant radionuclides, the provisional regulatory values and current criteria were set considering contributions from other radionuclides, such as strontium-90, to improve the effectiveness of monitoring tests [10,11,12,13,14,15].

Based on the current criteria and the guideline for the monitoring inspection of radioactive materials in foods, the monitoring test for radioactive cesium by the local governments is conducted around 17 prefectures in eastern Japan, including the Fukushima Prefecture [15,16]. Foods containing radioactive substances exceeding the standard limits are recalled and disposed. Furthermore, when the regional spread of foods exceeding the standard limits is identified from the monitoring test, the food distribution is restricted in the corresponding prefecture (or in small areas within the prefecture). Thus, rigorous actions are implemented to ensure that foodstuffs containing radioactive substances exceeding the current criteria are not distributed. In Japan, various food safety measures have been taken over the past decade [17].

Since the establishment of safety measures for radionuclides in foodstuffs in 2011, monitoring tests have been conducted using radiation-measuring instruments (e.g., a germanium semiconductor detector) to measure the radionuclide concentration in foods. Over 2,500,000 monitoring tests have been performed under the current criteria (Table 1) until fiscal year 2020 (FY2020) [15,18,19]. In a previous study, we reported the radiation dose owing to the ingestion of foodstuff and the effect of regulations using the accumulated monitoring results effectively as a preliminary study [20]. In this study, we estimated the longitudinal change in internal exposure dose under the current criteria for food safety to validate the effect of regulation using the same method as in our previous study [20]. Furthermore, as a new attempt, we estimated radiation dose considering the intake of edible wild vegetables. Although edible wild vegetables are valuable for local residents in many aspects (i.e., in consuming, gathering, selling, and sharing) [21], they can contain a relatively high concentration of radionuclides [15,22,23,24,25]. Therefore, the intake of wild vegetables should be reflected in the estimated radiation dose. However, nutrition surveys, such as the National Health and Nutrition Survey (NHNS) Japan, that provide food intake amount by food category do not specifically indicate the intake of wild vegetables. Therefore, the consumption of wild vegetables is not considered in contaminant (e.g., radionuclide)-related consumption surveys, such as total diet study (e.g., market basket survey). In this study, as a new challenge in exposure surveys, we estimated an exposure dose reflecting the intake of wild vegetables based on the data indicating the intake of wild vegetables obtained from a survey conducted in a previous study [26].

## 2. Materials and Methods

### 2.1. Subject for Evaluation

The radiation dose was estimated for five years (FY2012–FY2016) after applying the current criteria using the monitoring results throughout Japan.

### 2.2. Preparation of Data

#### 2.2.1. Data of the Monitoring Tests Results

Reports of test results released monthly on the MHLW website [18] were downloaded. The radioactivity concentration of cesium (Cs-134, Cs-137, and sum of Cs-134 and Cs-137) (Bq/kg) is presented according to the purchase day or sampling day, general food categories, food item names (3119, 2328, 2335, 1974, and 1728 items in FY2012, FY2013, FY2014, FY2015, and FY2016, respectively), and production area. The number and percentage of test results exceeding the criteria in each FY were analyzed. Additionally, the distribution of radioactivity concentration in mountain-based foodstuffs that sometimes have a high level of radionuclides (such as wild vegetables, wild animal meats, and mushrooms) was analyzed.

In the present study, we used the sum of radioactivity concentrations of Cs-134 and Cs-137 to calculate internal exposure dose. The downloaded results for which the total radioactivity concentration, purchase day, or sampling day could not be identified were excluded for data cleaning because they cannot be used for dose estimation. These results were classified according to FY based on the purchase day or sampling day. The database was constructed with cleaned test results using Microsoft Access 2019 (Microsoft Japan Co., Ltd., Tokyo, Japan).

For some food items, radioactivity concentration was adjusted to assume the state of “ready to eat”—a concept presented by CAC [7,27]. The radioactivity concentration of brown rice was set to a quarter based on previous studies [12,28]. Additionally, the radioactivity concentration of leaves of the plant that will be extracted and drunk was set to one-fiftieth based on a previous report [29]. However, radioactivity concentration was not adjusted for green tea leaves because green-tea-infused liquid is tested (i.e., the monitoring test result is generally listed as the value for the infused liquid) [30].

#### 2.2.2. Food Intake

The food intake amount in the reports of 2012 NHNS Japan was used [31]. The food intake (g/day) classified into 98 small classifications of foodstuffs is shown in this survey [32]. Table 2 presents 98 small classifications and the average intake shown in the survey with a small classification number (SCN). In this study, we used the average value of food intake for adult men and women (over 20 years old).

Because the intake of wild vegetables was included in the report of the survey of special tally work for the food intake frequency and intake, which was irregularly published in 2010 [26], we selected wild vegetable items from the report and added those intakes. Consequently, we treated 36 species (7.67 g/day of total intake, Table 2) as wild vegetables. The seasonal variations in intake were considered in the survey. Additionally, the intake volume of drinking water, which is not indicated in NHNS, was set as 2 L/day (Table 2), similar to the considerations in the current criteria [10]. Therefore, 99 types of foodstuffs (for dose estimation “without the reflection of intake of wild vegetables”) and 100 types of foodstuffs (“with the reflection of intake of wild vegetables”) were considered in the food intake data.

**Figure 1:**
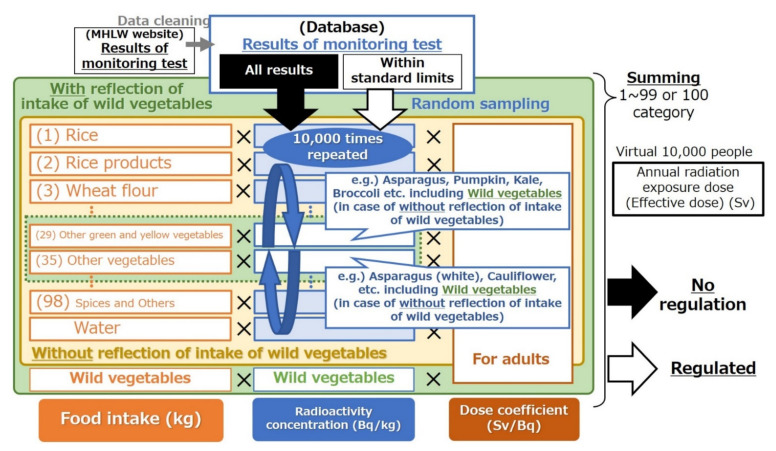
Schematic of data acquisition and dose calculation. Random sampling was individually repeated for the monitoring test results within the standard limits or for all results. The radiation exposure dose of virtual 10,000 persons was calculated as the multiplication product of the food intake in each food category, sampled radioactivity concentration, and dose coefficient. In radiation dose estimation without the reflection of intake of wild vegetables, wild vegetables were considered as “other green and yellow vegetables” or/and “other vegetables”. The number in parentheses denotes a small classification number in NHNS.

Similar to a previous study [20], the foodstuff item names in the test results were assigned to the 99 or 100 types of foodstuffs in the food intake data. Details of this methodology can be found in a previous study [20]. Wild vegetables, such as “bracken”, were classified into “other green and yellow vegetables” (SCN 29) or “other vegetables” (SCN 35) in dose estimation “without the reflection of intake of wild vegetables”. Additionally, wild vegetables in the test results were directly linked to the wild vegetables’ intake in dose estimation “with the reflection of intake of wild vegetables”, as shown in Figure 1.

#### 2.2.3. Dose Coefficient

To convert the radioactivity (Bq) of foodstuff consumed to radiation exposure dose (Sv), we used dose coefficients (dose conversion factor: DCF) for ingestion for an adult based on the ICRP publication 72 [33]. The DCF (Sv/Bq) values of Cs-134 and Cs-137 for an adult are 1.9 × 10^−8^ and 1.3 × 10^−8^, respectively. The physical half-lives of Cs-134 and Cs-137 are 2.06 and 30.2 years, respectively [34]. The weighted average DCF value for each FY was calculated using a decay rate based on half-lives of Cs-134 and Cs-137:

(1) $$\mathrm{Weighted}\;\mathrm{average}\;\mathrm{DCF}\;\left(\mathrm{Sv}/\mathrm{Bq}\right)=\left(1.9\cdot 10^{-8}\cdot DR_{134,y}+1.3\cdot 10^{-8}\cdot DR_{137,y}\right)/\left(DR_{134,y}+DR_{137,y}\right)$$

where *DR*_134,y_ and *DR*_137,y_ represent the decay rate of Cs-134 and Cs-137 in each fiscal year, respectively. The decay rate in each fiscal year was obtained considering physical half-life and elapsed time.

The weighted average DCF values for each FY are listed in Table 3. These weighted average DCF values were used to calculate radiation dose as the dose coefficient for the radioactivity of the sum of Cs-134 and Cs-137.

### 2.3. Data Acquisition and Dose Calculation

Figure 1 shows the schematic of data acquisition and dose calculation. The monitoring test results were randomly sampled for each food category (i.e., 99 or 100 classifications). Random sampling was individually conducted based on the following four methodologies: (1) using the data of test results within the standard limits (regulated) with the reflection of intake of wild vegetables, (2) using the results within the standard limits (regulated) without the reflection of intake of wild vegetables, (3) using all results (no regulation) with the reflection of intake of wild vegetables, and (4) using all results (no regulation) without the reflection of intake of wild vegetables. The internal exposure dose when the food distribution was restricted under the current criteria (assuming “regulated”) was estimated using the test results within the standard limits based on the assumption that food exceeding the limits is not distributed by rigorous regulations. However, all monitoring data were used to calculate the radiation dose when distribution restrictions were not considered (assuming “no regulation”). The methodology for radiation dose estimation using test results has been used previously. The radiation exposure dose under regulation measures was estimated using the test results within the criteria when the present standard limits were developed by the MHLW [10,35].

Annual internal exposure dose (committed effective dose) was obtained as the multiplication product of the food intake, total radioactivity concentration of Cs-134 and Cs-137 in each food category, and dose coefficient:

(2) $$\mathrm{Committed}\;\mathrm{effective}\;\mathrm{dose}\;\left(\mathrm{mSv}/\mathrm{year}\right)=365.24\cdot 10^{3}\cdot DCF\sum_{i=1}^{99\;\mathrm{or}\;100}I_{i}\cdot C_{i},$$

where *I_i_* represents the food intake (kg/day) in each classification and *C_i_* represents the radioactivity concentration of radioactive cesium (sum of Cs-134 and Cs-137) (Bq/kg) sampled randomly from each food classification.

The internal exposure dose of 10,000 virtual persons was calculated in each pattern by random sampling 10,000 times. As in a previous study [20], for the test results, where radioactive cesium was not detected (ND) (i.e., results below the limit of detection: LOD), the radioactivity concentration was given based on the ratio of the ND samples in each category to calculate the internal exposure dose [10,12,36]. Details regarding this rule can be referenced in the previous study [20].

## 3. Results

### 3.1. Analysis of the Monitoring Results

**Figure 2:**
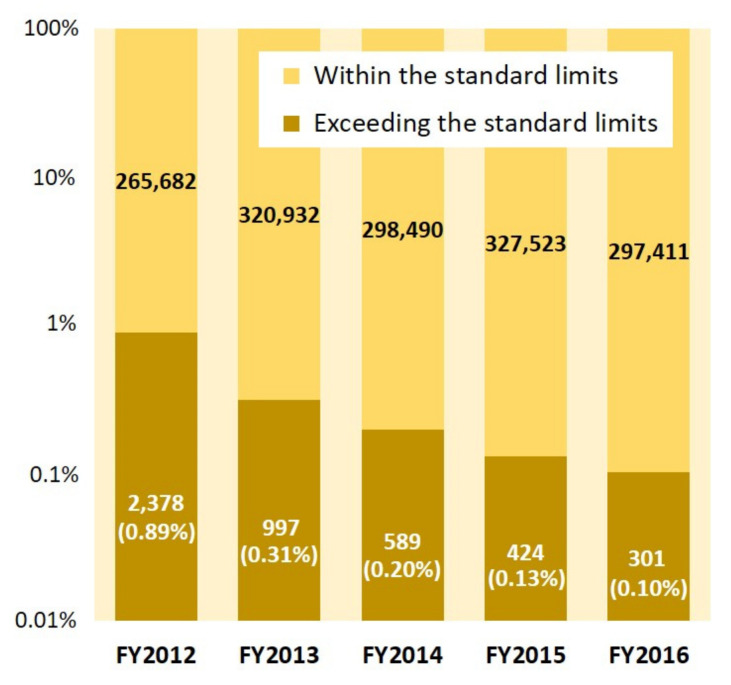
Number of results within the standard limits and the number of results exceeding the standard limits for all food categories in each fiscal year (FY). The percentage of results exceeding the standard limits is shown in parentheses. Since FYs of monitoring results were distinguished by the date of purchase or sampling in our research, and data cleaning was performed, totaled results may not match other data (e.g., data published by MHLW).

Figure 2 shows the number of all monitoring results and the number of results exceeding the standard limits with violation rate in each FY. The percentage of results exceeding the standard limits was approximately 1% in FY2012. Thereafter, the violation rate showed a downward trend; it decreased to 0.10% in FY2016.

### 3.2. Distribution of Radioactivity Concentration of Wild Vegetables, Wild Animal Meats, and Mushrooms

**Figure 3:**
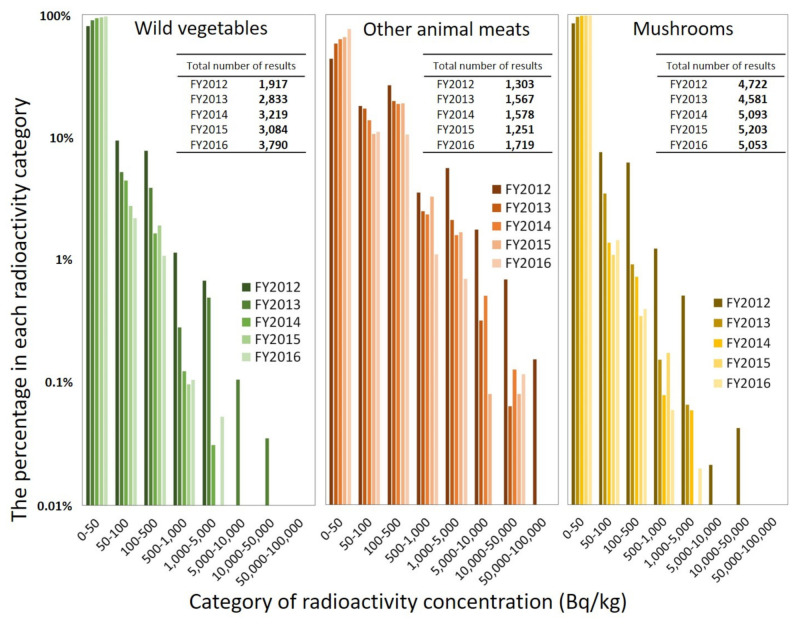
Percentage of monitoring test results for wild vegetables, other animal meats, and mushrooms in each radioactivity category. As it is often impossible to determine from the test results, natural and cultivated products are counted without distinction. Nonplotted segments (i.e., less than 0.01%) were zero percent.

Because this study considers wild vegetables as one of the themes, the distribution of the radioactivity concentration of mountain-based foodstuffs, such as wild vegetables, wild animal meats (SCN 64), and mushrooms (SCN 46) as one of the foods that sometimes include a high level of radionuclides, was analyzed (Figure 3). In terms of the number of test results by FY, the number of results in FY2012 was lower than that in other FYs, particularly for wild vegetables. Some results exceeded the standard limit (i.e., 100 Bq/kg for general food) for all three types of foods. However, there were fewer samples of wild vegetables and mushrooms with high radioactivity concentrations than those in the case of animal meats. The median radioactivity concentration of wild vegetables, wild animal meats, and mushrooms in FY2012 were 15.0, 66.0, and 18.0 Bq/kg, respectively. The 95th percentile of the radioactivity concentration of wild vegetables, wild animal meats, and mushrooms in FY2012 were 222, 2500, and 180 Bq/kg, respectively. The 99th percentiles of the radioactivity concentration of wild vegetables, wild animal meats, and mushrooms in FY2012 were 836, 9000, and 756 Bq/kg, respectively. In this way, the degree of radioactivity concentrations of wild vegetables and mushrooms were lower than in other animal meats. Similar results were obtained in other FYs as well.

Additionally, the distribution of all the three types of foods shifted to the lower-concentration side with time. Each percentile value was the highest in FY2012 among the five years.

### 3.3. Estimated Internal Exposure Dose

**Figure 4:**
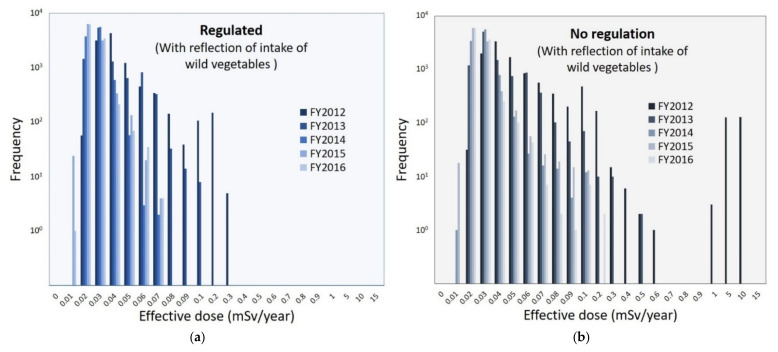
Radiation exposure doses of virtual 10,000 persons with the reflection of intake of wild vegetables in each fiscal year (FY): (**a**) regulation and (**b**) no regulation. The frequency of nonplotted segments (i.e., with a frequency of less than 10^0^) was zero.

Figure 4 shows the distribution of the estimated radiation dose of virtual 10,000 persons with the reflection of intake of wild vegetables in each FY. In the estimation with all test results (assumed to be “no regulation”) for FY2012, there were cases in which the committed effective dose significantly exceeded 1 mSv/year (the maximum dose was 12.0 mSv/year, Figure 4b). However, in the estimation with results within the standard limits (assumed to be “regulated”) for FY2012, the committed effective dose was considerably below 1 mSv/year in all cases (Figure 4a). Furthermore, there was no case that exceeded 1 mSv/year for FY2013–2016, regardless of the regulation. The mode (peak) of distribution shifted to the lower radiation dose as the years progressed.

### 3.4. Longitudinal Change of Internal Exposure Dose

**Figure 5:**
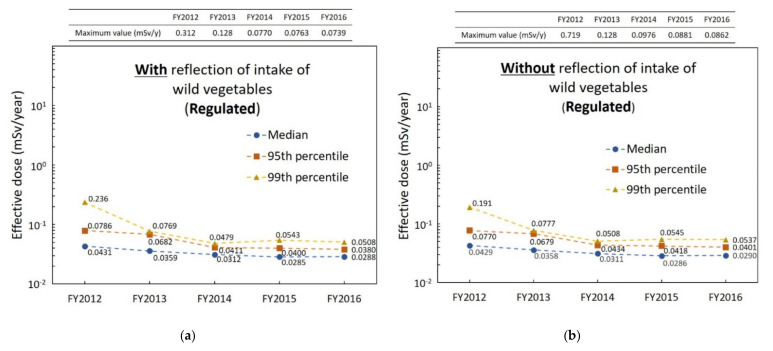
Longitudinal change of internal exposure dose for the case assumed to be “regulated”: (**a**) with and (**b**) without the reflection of intake of wild vegetables.

Figure 5 shows the median as well as 95th and 99th percentiles of the internal exposure dose of virtual 10,000 persons for the case of “regulated” for each FY. Figure 5a,b show the percentiles with and without the reflection of intake of wild vegetables, respectively. There was no significant difference between the effective dose of each percentile with and without the reflection of intake of wild vegetables for each FY. Each percentile of the effective dose gradually decreased over the years, and the values of each percentile were close in FY2016.

ICRP advocates that the radiation dose of the 95th percentile is defined as that received by a “representative person” [37]. It is thought that if the radiation dose of the 95th percentile in a certain group is below the adopted criterion (e.g., 1 mSv/year in this case), the group is protected. In this study, the effective dose of the representative person (i.e., the value of 95th percentile) was below 1 mSv/year for the case of “regulated” with and without the reflection of intake of wild vegetables were for all FYs. The radiation doses of the 99th percentile were also below 1 mSv/year.

**Figure 6:**
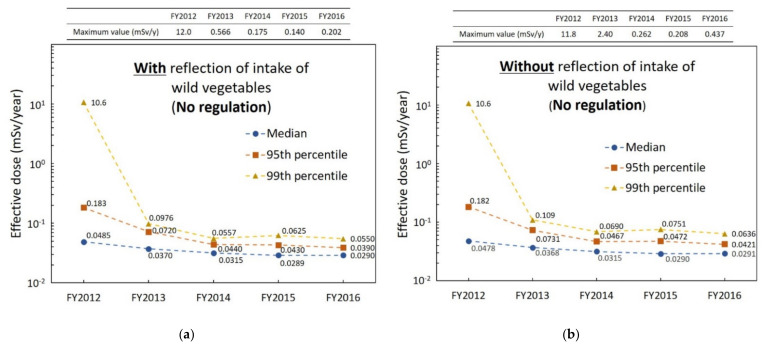
Longitudinal change of internal exposure dose in the case assumed to be “no regulation”: (**a**) with and (**b**) without the reflection of intake of wild vegetables.

Figure 6 shows the median as well as 95th and 99th percentiles of the internal exposure dose of virtual 10,000 persons in the case of “no regulation” in each FY. Figure 6a,b show each percentile with and without the reflection of intake of wild vegetables, respectively. There was no significant difference between the effective dose of the median with and without the reflection of intake of wild vegetables for each FY. However, the 95th and 99th percentiles (especially 99th percentile) of effective dose from FY2013 to FY2016 without reflection of intake of wild vegetables were slightly higher than those with the reflection of intake of wild vegetables (there was no significant difference in FY2012). Furthermore, in contrast to the case with the reflection of intake of wild vegetables, the maximum value without reflection of the intake of wild vegetables in FY2013 exceeded 1 mSv/year.

## 4. Discussion

Although more than ten years have passed since the FDNPP accident, consumer awareness surveys have shown that interest in food safety for radionuclides may still be high in some cases [38,39]. Therefore, it is essential to verify regulations, such as setting standard limits and monitoring inspections [14,40], which should be done on an ongoing basis. Additionally, as in the case of the dose assessment of hunters who subsist on the products from the forest ecosystem after the Chernobyl nuclear power plant accident [41], a study regarding the radiation dose of residents who consume a high amount of local food in the region is also necessary. Although not limited to the radiation emergency, interest in radioactive materials in food and drinking water is internationally high [42], requiring multipronged research. In this study, we estimated the longitudinal change of internal exposure dose under the current food regulations, considering the intake of wild vegetables, which is a valuable item for local residents.

As shown in Figure 2, the characteristics of monitoring test results for all food categories show that the violation rate about five years after the FDNPP accident was approximately one-tenth compared to that in the first year where the current criteria were applied. Additionally, we have previously reported the number and percentage of monitoring test results according to the radioactivity concentration in each food rough category [20]; it was shown that some samples in the categories of drinking water, agricultural products, animal products, fishery products, wild animal meats, and other food exceeded the standard limits for FY2012. Although high radioactivity concentration was found in many samples under the wild animal meat category, there are no samples exceeding the standard limit in the milk or infant food category for FY2012. In the previous study [20], we have also stated that the samples exceeding the standard limit decreased considerably, and no sample exceeded the standard limit for the drinking water, milk, or infant food and animal product categories for FY2016. However, some samples in the wild animal meat and agricultural product categories still demonstrated high radioactivity concentrations. Based on the above, although the percentage of samples exceeding the standard limit is small (less than 1%), it is suggested that the impact of the accident on foodstuff has become substantially small a few years after the accident, except for a few food items. This is because of the decrease of radionuclides in foods due to the radioactive decay, reduction measures, etc. In terms of radioactive decay, Cs-134 decreases more quickly than Cs-137 due to its short half-life. This is also observed in the change of the weighted average DCF for the sum of Cs-134 and Cs-137. The weighted average DCFs become slightly smaller over time since the proportion of Cs-137, whose DCF is smaller than that of Cs-134, becomes larger (Table 3). This was reflected in the internal radiation doses, as each percentile of the committed effective dose decreased with the passage of years (Figure 5 and Figure 6). Similarly, the previous study [20] has provided a breakdown of high cesium intake by small classification in addition to the data on radioactivity concentration in foods. Additionally, the number of monitoring tests of wild vegetables has tended to increase since FY2013 (Figure 3), which seems to reflect an enhancement of the inspection system.

In the radiation dose estimation for FY2012 with all test results (“no regulation”), there were some cases that exceeded the committed effective dose of 1 mSv/year significantly (>10 mSv). In our previous study, it was stated for the high radiation dose that this high value is mainly attributed to the high radionuclide intake through the consumption of other beverages [20]. Specifically, the beverages were powdered beverages made of plant leaves (SCN 91 in Table 2). However, the powdered beverage is commonly intended to be consumed as a dilute solution or extracted infusion. Furthermore, it is unlikely that the same powdered beverage keeps being consumed. Therefore, the high radiation dose was perhaps overestimated. The assumption that the same sample is repeatedly consumed is one of the limitations of the total diet study.

Meanwhile, in the estimation for FY2012 with results within the standard limits (assumed to be “regulated”), the committed effective dose was considerably below 1 mSv/year in all cases. Even in the case of “no regulation”, the 95th percentile of effective dose, a “representative person” dose, was sufficiently below 1 mSv/year. Comparing the radiation doses for “regulated” and “no regulation” cases for FY2012, the higher the percentile of radiation dose, the smaller the “regulated” radiation dose than that of “no regulation.” Furthermore, the median, as well as the 95th and 99th percentiles of internal exposure dose in four patterns (i.e., regulated or no regulation, with or without the reflection of the intake of wild vegetables), were considerably less than 1 mSv/year for FY2013–2016. The values gradually decreased over the years. Therefore, it was considered that the effect of regulation was particularly large in FY2012, and appropriate regulations have ensured food safety in Japan since the time the current criteria were adopted. Moreover, even when the provisional regulation values were established based on the effective dose of 5 mSv/year and applied in 2011, the radiation exposure dose from radioactive cesium was well below 1 mSv/year [35,43].

Next, we discuss the cases where the food intake of wild vegetables were considered and where they were not. In the case of “regulated”, there was no significant difference between the effective dose of each percentile with and without the reflection of intake of wild vegetables for each FY. As mentioned in the previous section, although wild vegetables can contain relatively high concentrations of radioactive cesium, the degree of radioactivity concentration is not very high, as shown in Figure 3. Additionally, the food intake of wild vegetables is not so large compared to other vegetables, as shown in Table 2. As a result, it was thought that the internal exposure dose resulting from the consumption of wild vegetables was not high. Therefore, it was suggested that the radiation doses with and without the reflection of intake of wild vegetables were similar. However, in the case of “no regulation”, although there was no significant difference between the effective dose of the median with and without the reflection of intake of wild vegetables for each FY, the 95th and 99th percentiles (especially 99th percentile) of effective dose without the reflection of intake of wild vegetables were slightly higher than those with the reflection for FY2013–2016. In the dose estimation of “no regulation”, because the monitoring test results of wild vegetables were picked up as general vegetables (SCN 29 or 35), the food intake of wild vegetables (7.67 g/day) was considered to be that of general vegetable (35.9 or 48.3 g/day). Therefore, when the monitoring test result of high radioactivity concentration (e.g., 99th percentile was 836 Bq/kg for FY2012) is picked up in random sampling, the internal radiation dose resulting from the consumption of wild vegetables was overestimated. Thus, it was suggested that the high percentile of effective dose from FY2013 to FY2016 (“no regulation”) without the reflection of intake of wild vegetables were slightly higher than those with the reflection. By considering the intake of wild plants, it was considered that overestimation could be prevented, and a more precise dose estimation could be achieved. Thus, better data for consuming wild vegetables, which grow in the wild and are difficult to manage during cultivation, were provided in this study.

As mountain-based foodstuffs, wild mushrooms are of significance to local residents, as well as wild vegetables. Although some samples exceeded the standard limit, the distribution of radioactivity concentration in mushrooms was on the low side compared to other animal meats (Figure 3). Furthermore, the radioactivity level and amount of food consumption are important factors in radiation dose estimation [20]. Because the intake of mushrooms is not so large (SCN46; 17.2 g/day in Table 2), the radiation dose resulting from consuming mushrooms is not considered to be large, as is the case with wild vegetables. This finding is thought to be beneficial to the local residents. Because we used the average food intake, biases of individual dietary habits (e.g., in case of high intake) could not be reflected. Therefore, this study does not provide dose estimates that reflect the habitual intake of individuals (as described in the above section, only the intake of wild vegetables reflects the seasonal variation in intake), which is one of the limitations. In future studies, we would like to explore a method of dose estimation that reflects habitual intake based on a previous study [44].

In the estimation of internal radiation dose resulting from the consumption of radionuclides in foods, detailed research using the actual distribution of foods (market basket study) has been conducted by the MHLW. The study of the MHLW also indicated that the radiation exposure dose resulting from the ingestion of radioactive cesium was considerably smaller than the reference level of 1 mSv/year [45]. However, the estimated radiation dose in this study was generally larger than that estimated in the market basket study. For instance, the internal exposure doses for FY2012 were evaluated to be 0.0009–0.0057 mSv/year and 0.0008–0.0071 mSv/year in the market basket study [46,47]. This is because of the large LOD in the monitoring tests [20]. Because the monitoring test requires convenience, an inspection is performed with a large LOD, which depends on the measurement time and sample volume compared to the market basket study. Therefore, the internal exposure dose in this study would be overestimated. However, we believe it is not a major problem in evaluating the effect of regulations as the radiation dose “regulated” and “no regulation” were estimated under the same conditions in this study.

## 5. Conclusions

In this study, a longitudinal change in the internal exposure dose under the current food regulation was evaluated using a number of accumulated monitoring results, taking into account the intake of wild vegetables. The effect of regulation was particularly large for FY2012. Subsequently, there was no noticeable difference in the doses with and without the regulations, and they decreased as the years passed. The internal radiation doses under regulation in each FY were substantially smaller than the reference level of 1 mSv/year. Additionally, it was considered that the overestimation for the high percentile assuming no regulation could be prevented by reflecting the intake of wild vegetables, and a more plausible dose estimation could be achieved. Thus, it was confirmed that food safety with respect to radionuclides in Japan was ensured through the implementation of appropriate measures, even taking into account wild vegetables that grow in the wild and are difficult to manage during cultivation.

## Figures and Tables

**Table 1:** Current criteria for radioactive cesium (sum of Cs-134 and Cs-137) in foods in Japan (established on 1 April 2012).

Category	Limit (Bq/kg)
Drinking water	10
Milk	50
General food	100
Infant food	50

**Table 2:** Food category and intake used in this study. Here 1–98 corresponds to small classifications shown in NHNS.

No.	Small Classification	Intake (g/day)	No.	Small Classification	Intake (g/day)	No.	Small Classification	Intake (g/day)
1	Rice	328	35	Other vegetables	48.3	69	Other meats and Processed products	0.00898
2	Rice products	4.18	36	Vegetable juices	12.7	70	Eggs	34.1
3	Wheat flour	3.56	37	Leaf pickles	3.82	71	Milk	60.6
4	Breads (except Japanese buns)	33.1	38	Other pickles	8.44	72	Cheeses	2.48
5	Japanese buns	4.39	39	Strawberries	0.0889	73	Fermented milk and Lactic acid bacteria beverages	30.6
6	Japanese noodles and Chinese noodles	43.0	40	Citrus fruits	22.7	74	Other dairy products	6.18
7	Precooked noodles	5.13	41	Bananas	15.9	75	Others (in Milks)	0
8	Macaroni and Spaghetti	10.7	42	Apples	21.9	76	Butters	1.00
9	Other wheat products	5.38	43	Other fruits	39.9	77	Margarines	1.25
10	Buckwheat and Buckwheat products	6.52	44	Jams	1.29	78	Vegetable fats and oils	8.05
11	Corn and Corn products	0.388	45	Fruit juices and Fruit juice beverages	8.03	79	Animal fats	0.108
12	Other cereals	2.04	46	Mushrooms	17.2	80	Others (in Fats and Oils)	0.00779
13	Sweet potatoes and Sweet potato products	6.97	47	Algae	10.5	81	Traditional confectioneries	11.7
14	Potatoes and Potato products	25.7	48	Horse mackerels and Sardines	9.14	82	Cakes, Buns, and Pastries	6.71
15	Other potatoes and Potato products	19.8	49	Salmons and Trouts	5.54	83	Biscuits	1.62
16	Starches and Starch products	1.98	50	Sea breams and Righteye flounders	5.69	84	Candies	0.165
17	Sugars and Sweeteners	6.74	51	Tunas, Marlins, and Swordfishes	4.77	85	Others (in Confectioneries)	4.67
18	Soybean (whole beans) and its products	1.28	52	Other fishes	9.02	86	Sake	11.3
19	*Tofu* (Bean curd)	35.5	53	Shellfishes	3.16	87	Beer	76.7
20	*Abura-age*	8.18	54	Cephalopods	4.21	88	Wines, Spirits, and Others	36.1
21	Natto (Fermented soybeans)	8.04	55	Prawns, Shrimps, and Crabs	4.73	89	Teas	296
22	Other soybean products	7.20	56	Seafood (salted, semi-dried, and dried)	15.8	90	Coffees and Cocoas	151
23	Other pulses and Pulse products	1.40	57	Seafood (canned)	2.31	91	Others (in Other beverages of Beverages)	102
24	Nuts and Seeds	2.24	58	Seafood (Tsukudani)	0.294	92	Sauces	1.88
25	Tomatoes	15.2	59	Seafood (Fish paste products)	10.2	93	*Shoyu*: soy sauces	14.2
26	Carrots	20.3	60	Fish hams and Sausages	0.729	94	Edible salts	1.35
27	Spinach	14.7	61	Beefs	14.5	95	Mayonnaise	2.89
28	Sweet peppers	4.86	62	Porks	33.7	96	Miso	11.6
29	Other green and yellow vegetables	35.9	63	Hams and Sausages	12.6	97	Other seasonings	63.4
30	Cabbages	28.6	64	Other animal meats	0.361	98	Spices and Others	0.333
31	Cucumber	9.68	65	Chickens	23.9		Drinking water ^1^	2000
32	*Daikon* (Japanese radishes)	32.4	66	Others (in Poultries of Meats)	0.0751		Wild vegetables ^2^	7.67
33	Onions	31.4	67	Offals	1.52			
34	Chinese cabbage	20.4	68	Whale meat	0.0354			

^1^ The intake of drinking water, which is not indicated in NHNS, was set as 2 L/day. ^2^ The intake of wild vegetables was taken from the data of other surveys [26] than the NHNS and summed up.

**Table 3:** Dose coefficients/dose conversion factors for the total radioactivity concentration of Cs-134 and Cs-137 for fiscal years (FYs) 2012–2016.

	FY2012	FY2013	FY2014	FY2015	FY2016
(Sv/Bq)	1.55 × 10^−8^	1.51 × 10^−8^	1.47 × 10^−8^	1.43 × 10^−8^	1.40 × 10^−8^

## Data Availability

Monitoring results used in this study is available on the MHLW website.

## References

[1] Nuclear and Industrial Safety Agency (at that Time). https://warp.da.ndl.go.jp/info:ndljp/pid/8422823/www.meti.go.jp/press/2011/06/20110606008/20110606008-2.pdf.

[2] Ministry of Health, Labour and Welfare (2011). Notice No. 0317 Article 3 of the Department of Food Safety. https://www.mhlw.go.jp/stf/houdou/2r9852000001558e-img/2r98520000015av4.pdf.

[3] Ministry of Health, Labour and Welfare (2011). Press Release: Handling of Food Contaminated by Radioactivity. https://www.mhlw.go.jp/english/topics/foodsafety/dl/food-110317.pdf.

[4] Ministry of Health, Labour and Welfare (2012). Notice No. 0315 Article 1 of the Department of Food Safety. https://www.mhlw.go.jp/english/topics/2011eq/dl/food-120821_1.pdf.

[5] International Commission on Radiological Protection (1999). Protection of the public in situations of prolonged radiation exposure, ICRP Publication 82. Ann. ICRP.

[6] Codex Alimentarius Commission General Standard for Contaminants and Toxins in Food and Feed, CXS 193-1995. http://www.fao.org/fao-who-codexalimentarius/sh-proxy/en/?lnk=1&url=https%253A%252F%252Fworkspace.fao.org%252Fsites%252Fcodex%252FStandards%252FCXS%2B193-1995%252FCXS_193e.pdf.

[7] Codex Alimentarius Commission (2011). Fact Sheet: Codex Guideline Levels for Radionuclides in Foods Contaminated Following a Nuclear or Radiological Emergency. http://www.fao.org/fileadmin/user_upload/agns/pdf/codex_guideline_for_radionuclitide_contaminated_food.pdf.

[8] International Atomic Energy Agency (2014). IAEA Safety Standards Series No. GSR Part 3: Radiation Protection and Safety of Radiation Sources: International Basic Safety Standards.

[9] International Atomic Energy Agency (2016). IAEA-TECDOC-1788: Criteria for Radionuclide Activity Concentrations for Food and Drinking Water.

[10] Pharmaceutical Affairs and Food Sanitation Council Report on the Task Force on the Countermeasures against Radioactive Materials (Provisional Translation). https://www.mhlw.go.jp/stf/shingi/2r98520000023nbs-att/2r98520000023ng2.pdf.

[11] Iwaoka K. (2016). The Current Limits for Radionuclides in Food in Japan. Health Phys..

[12] Terada H., Yamaguchi I., Shimura T., Svendsen E.R., Kunugita N. (2018). Regulation values and current situation of radioactive materials in food. J. Natl. Inst. Public Health.

[13] Merz S., Shozugawa K., Steinhauser G. (2015). Analysis of Japanese radionuclide monitoring data of food before and after the Fukushima nuclear accident. Environ. Sci. Technol..

[14] Osanai M., Kudo K., Iwaoka K., Yamaguchi I., Tsushima M., Saito Y., Hosokawa Y. (2017). Verification of the assumption on contribution ratio to the reference level from each radionuclide in seafood to derive criteria for radionuclide activity concentrations for food in the existing exposure situation regarding the Fukushima Dai-ichi Nuclear Power Plant accident. Radioisotopes.

[15] Ministry of Health, Labour and Welfare Radioactive Materials in Foods-Current Situation and Protective Measures. https://www.mhlw.go.jp/english/topics/2011eq/dl/food-130926_1.pdf.

[16] The Nuclear Emergency Response Headquarters Concepts of Inspection Planning and the Establishment and Cancellation of Items and Areas to Which Restriction on Distribution and/or Consumption of Foods Concerned Applies, 2021 Revision. https://www.mhlw.go.jp/english/topics/2011eq/dl/food_revision_26%20March_2021.pdf.

[17] Yamaguchi I., Terada H., Shimura T., Yunokawa T., Ushiyama A. (2021). Measures taken to ensure radiation safety of food after the accident at TEPCO’s Fukushima Daiichi Nuclear Power Station -Summary of measures implemented over 10 years. J. Natl. Inst. Public Health.

[18] Ministry of Health, Labour and Welfare Levels of Radioactive Materials in Foods Tested in Respective Prefectures. https://www.mhlw.go.jp/english/topics/2011eq/index_food_radioactive.html.

[19] National Institute of Public Health Database of Radioactive Substances in Foods. http://www.radioactivity-db.info/.

[20] Osanai M., Hirano D., Mitsuhashi S., Kudo K., Hosokawa S., Tsushima M., Iwaoka K., Yamaguchi I., Tsujiguchi T., Hosoda M. (2021). Estimation of Effect of Radiation Dose Reduction for Internal Exposure by Food Regulations under the Current Criteria for Radionuclides in Foodstuff in Japan Using Monitoring Results. Foods.

[21] Matsuura T., Hayashi M., Sugimura K., Tanaka N., Miyamoto A. (2013). Ecosystem services valuation of harvesting edible wild plants/mushrooms–A case study in Tadami Town, Fukushima Prefecture. Jpn. J. For. Plann..

[22] Kiyono Y., Akama A. (2013). Radioactive cesium contamination of edible wild plants after the accident at the Fukushima Daiichi Nuclear Power Plant. Jpn. J. For. Environ..

[23] Kiyono Y., Komatsu M., Akama A., Matsuura T., Hiroi M., Iwaya M., Futamoto T. (2018). The transfer of radiocesium released in the 2011 Fukushima Daiichi Nuclear Power Station accident to leaves of wild Osmunda japonica, an edible fern. Bull. FFPRI.

[24] Kiyono Y., Akama A. (2019). Cesium-137 food-processing factors and food-processing retention factors of 11 organs and 10 edible wild plant species from Japan: Recipes for long-term preservation reduced the radiocesium mass the most. Bull. FFPRI.

[25] Nabeshi H., Tsutsumi T., Uekusa Y., Matsuda R., Akiyama H., Teshima R., Hachisuka A. (2016). Effects of cooking process on the changes of concentration and total amount of radioactive caesium in beef, wild plants and fruits. Radioisotopes.

[26] Ministry of Health, Labour and Welfare List of Reports (provisional translation). https://www.mhlw.go.jp/stf/seisakunitsuite/bunya/kenkou_iryou/shokuhin/syokuten/houkokusyo/index.html.

[27] (2015). Theelen, R. (Chair of the Electronic Working Group, Netherlands); Osanai, M (Co-Chair of the Electronic Working Group, Japan). Discussion Paper on Radionuclides. Codex Committee on Contaminants in Foods 9th Session, Joint FAO/WHO Food Standards Programme. https://www.fao.org/fao-who-codexalimentarius/sh-proxy/en/?lnk=1&url=https%253A%252F%252Fworkspace.fao.org%252Fsites%252Fcodex%252FShared%2BDocuments%252FArchive%252FMeetings%252FCCCF%252Fcccf9%252Fcf09_14e.pdf.

[28] Sato M., Fujimura S., Fujita S., Suzuki Y., Sakuma Y., Owadad M. (2013). Distributions of radiocesium in rice plant and brown rice, and change in radiocesium concentration in rice grain by cooking. Bull. Fukushima Agric. Technol. Cent..

[29] Tsutsumi T., Hachisuka A. Report of Health and Labour Sciences Research Grants, FY2011. https://mhlw-grants.niph.go.jp/system/files/2011/114031/201131057A/201131057A0003.pdf.

[30] Ministry of Health, Labour and Welfare (2012). Notice No. 0315 Article 4 of the Department of Food Safety. https://www.mhlw.go.jp/english/topics/2011eq/dl/food-120821_2.pdf.

[31] Ministry of Health, Labour and Welfare Report on the 2012 National Health and Nutrition Survey Japan (Provisional Translation). https://www.mhlw.go.jp/bunya/kenkou/eiyou/h24-houkoku.html.

[32] National Institute of Health and Nutrition (2012). Outline of the National Health and Nutrition Survey (NHNS) Japan. https://www.nibiohn.go.jp/eiken/english/research/pdf/nhns2012.pdf.

[33] International Commission on Radiological Protection (1995). Age-dependent doses to the members of the public from intake of radionuclides-part 5 compilation of ingestion and inhalation coefficients, ICRP Publication 72. Ann. ICRP.

[34] Japan Radioisotope Association (2011). Radioisotope Pocket Data Book.

[35] Pharmaceutical Affairs and Food Sanitation Council https://www.mhlw.go.jp/shinsai_jouhou/dl/hibakusenryousuikei_02.pdf.

[36] World Health Organization (2006). GEMS/Food Total Diet Studies. https://www.who.int/foodsafety/publications/chem/TDS_Beijing_2006_en.pdf.

[37] International Commission on Radiological Protection (2006). The optimisation of radiological protection-broadening the process, ICRP Publication 101b. Ann. ICRP.

[38] Consumer Affairs Agency, Government of Japan (2021). Food and Radiation Q&A 15th Edition. https://www.caa.go.jp/disaster/earthquake/understanding_food_and_radiation/material/assets/consumer_safety_cms203_210721_01.pdf.

[39] Consumer Affairs Agency, Government of Japan (2013). Food and Radiation Q&A 8th Edition. https://www.caa.go.jp/disaster/earthquake/understanding_food_and_radiation/material/pdf/130902_food_qa_en.pdf.

[40] Yamaguchi I., Takahashi H. (2021). Estimating of internal radiation doses due to food consumption and its reduction applying the food regulation after the Fukushima nuclear accident using national food-monitoring data. J. Natl. Inst. Public Health.

[41] Tondel M., Rääf C., Wålinder R., Mamour A., Isaksson M. (2017). Estimated lifetime effective dose to hunters and their families in the three most contaminated counties in Sweden after the Chernobyl nuclear power plant accident in 1986–A pilot study. J. Environ. Radioact..

[42] International Atomic Energy Agency Technical Meeting on Radionuclides in Food and Drinking Water in non-emergency Situations. https://www.iaea.org/events/evt1904248.

[43] Hachisuka A., Matsuda R., Tsutsumi T., Igarashi A. Report of Health and Labour Sciences Research Grants, FY2011. https://mhlw-grants.niph.go.jp/system/files/2011/114031/201131057A/201131057A0004.pdf.

[44] Yokoyama T. (2013). Theory and application of statistical methods to estimate the distribution of usual intakes of a nutrient in a population: For the appropriate use of dietary reference intakes. Jpn. J. Nutr. Diet..

[45] Nabeshi H., Tsutsumi T., Imamura M., Uekusa Y., Hachisuka A., Matsuda R., Teshima R., Akiyama H. (2020). Continuous estimation of annual committed effective dose of radioactive cesium by market basket study in Japan from 2013 to 2019 after Fukushima Daiichi Nuclear Power Plant accident. Food Saf..

[46] Ministry of Health, Labour and Welfare Annual radiation dose from radionuclides in foods (September to October 2012) (provisional translation). https://www.mhlw.go.jp/stf/houdou/2r98520000034z6e.html.

[47] Ministry of Health, Labour and Welfare Annual radiation dose from radionuclides in foods (February to March 2013) (provisional translation). https://www.mhlw.go.jp/stf/houdou/0000032135.html.

